# The social network around influenza vaccination in health care workers: a cross-sectional study

**DOI:** 10.1186/s13012-016-0522-3

**Published:** 2016-11-24

**Authors:** Anna Llupià, Joaquim Puig, Guillermo Mena, José M. Bayas, Antoni Trilla

**Affiliations:** 1 Hospital Clínic-Universitat de Barcelona-ISGlobal, C/ Villarroel 170, 08036 Barcelona, Spain; 2 Department of Mathematics, Universitat Politècnica de Catalunya, Diagonal 647, 08028 Barcelona, Spain

**Keywords:** Influenza vaccination, Social network, Health care workers

## Abstract

**Background:**

Influenza vaccination coverage remains low among health care workers (HCWs) in many health facilities. This study describes the social network defined by HCWs’ conversations around an influenza vaccination campaign in order to describe the role played by vaccination behavior and other HCW characteristics in the configuration of the links among subjects.

**Methods:**

This study used cross-sectional data from 235 HCWs interviewed after the 2010/2011 influenza vaccination campaign at the Hospital Clinic of Barcelona (HCB), Spain. The study asked: “Who did you talk to or share some activity with respect to the seasonal vaccination campaign?” Variables studied included sociodemographic characteristics and reported conversations among HCWs during the influenza campaign. Exponential random graph models (ERGM) were used to assess the role of shared characteristics (homophily) and individual characteristics in the social network around the influenza vaccination campaign.

**Results:**

Links were more likely between HCWs who shared the same professional category (OR 3.13, 95% CI = 2.61–3.75), sex (OR 1.34, 95% CI = 1.09–1.62), age (OR 0.7, 95% CI = 0.63–0.78 per decade of difference), and department (OR 11.35, 95% CI = 8.17–15.64), but not between HCWs who shared the same vaccination behavior (OR 1.02, 95% CI = 0.86–1.22). Older (OR 1.26, 95% CI = 1.14–1.39 per extra decade of HCW) and vaccinated (OR 1.32, 95% CI = 1.09–1.62) HCWs were more likely to be named.

**Conclusions:**

This study finds that there is no homophily by vaccination status in whom HCWs speak to or interact with about a workplace vaccination promotion campaign. This result highlights the relevance of social network analysis in the planning of health promotion interventions.

**Electronic supplementary material:**

The online version of this article (doi:10.1186/s13012-016-0522-3) contains supplementary material, which is available to authorized users.

## Background

Although influenza vaccination is widely recommended to reduce the burden of influenza disease in patients and health care workers (HCWs), coverage remains low in many health facilities [1]. Many health promotion campaigns aimed at modifying health behaviors, such as influenza vaccination uptake, promote discussion among the subjects of the campaign. However, the results of fostering participation are not always in alignment with the campaign objective [2], for instance, increasing coverage. It is therefore interesting to consider how the health behavior in question affects the relations around the campaign. A suitable framework for this question is social network analysis (SNA) [3], a tool which is becoming increasingly popular in health-related issues [4] and has not been applied to vaccination campaigns in HCWs. There are increasing numbers of studies combining different types of social networks and health [5–7], with scope for possible network interventions [8]. In health care settings [9–11], these have been used to analyze hierarchies and positions in the network [12, 13], their relationship with hospitalization costs and readmission rates [14], information seeking [15, 16], the adoption of best practices [17], and homophily in advice and friendship networks, the tendency of individuals to associate and bond with similar others [18, 19]. Social networks may play a role in the adoption of health behaviors and practices [20], and homophily, in particular, has been shown to have an effect [21, 22]. The association between positions in the social network and decisions about vaccination [23] has been studied in parental vaccination decisions [24, 25], polio vaccine refusers [26], and high school [27] and medical students [28]. HCWs seem to be influenced by perceived pressure from colleagues to receive influenza vaccination [29]. To our knowledge, this is the first study to assess the relationship between the influenza vaccination status and the social network of HCWs, defined by conversations around the campaign.

After three influenza vaccination campaigns for HCWs at the Hospital Clínic of Barcelona (HCB) that fostered participation [30, 31], the present study was conducted to describe to whom HCWs speak or interact with around a campaign. In particular, we assessed whether HCWs were more likely to discuss vaccination and the campaign with peers with the same vaccination behavior. In addition, given that vaccination is a behavior which is not easily visible, we assessed the perception of vaccination behavior that HCWs have about their peers.

## Methods

### Study design and setting

The HCB is organized in departments which comprise various medical services. In November 2010, we interviewed HCWs from two of the 15 HCB departments (A and B), which include four medical services, in order to establish the social network around that season’s influenza vaccination campaign. This cross-sectional study was presented to HCWs and their representatives beforehand to increase participation. The payroll list was used as an indicator of the number of staff in the two departments. Four trained interviewers repeatedly visited all wards of the two departments during all working shifts (morning/afternoon/night/weekends) for 2 weeks asking HCWs gave names of their colleagues in order to answer the questions. The interviewers wrote down the identification number of each HCW searched for in the hospital payroll list. HCWs interviewed or cited by another HCW became a node of the network. When a HCW, the sender, named another HCW, the receiver, this citation defined a link. This is a directed network that allowed senders to be distinguished from receivers in links. HCWs interviewed were included if they belonged to one of the two departments, if they answered at least one interview question, and when demographic data was complete in the human resources (HR) data file. Cited HCWs were included in the study if data was complete, even if they did not belong to either of the two departments. Links were considered valid if both sender and receiver were included.

### Data sources and variables

We used three data sources. (1) The study survey. (2) Vaccination history from occupational health (OH) medical records. (3) HR data file.

1. The study survey (see Additional file 1) included two questions. The first, used to generate the network, was “Who did you talk to or share some activity with respect to the seasonal vaccination campaign?” The promotion campaign included vaccination in the wards, group photos after vaccination, fostering of promotion among peers, and informative sessions. These were defined as activities, all of which were voluntary. HCWs interviewed were asked to provide some information about each link and the contents of conversations. The second question asked for the names of other HCWs who they knew had been vaccinated.
2. The OH medical record included vaccination. HCWs not registered in the OH vaccination record were considered unvaccinated [32].
3. Demographic characteristics (sex, date of birth, professional category, department, and positions of responsibility) were obtained from the HR data file. Concordance between variables was created by cross-matching the masked ID which identifies every HCB HCW.

In included nodes with complete data, the following data were collected. Vaccination status, sex, and positions of responsibility (heads of service and coordinators in all professional categories) were considered dichotomously. Age was measured in years. Professional category included five categories: staff physicians, resident physicians, nurses, auxiliary nurses and orderlies, and administrative and technical staff. Department was analyzed in three categories (A, B, and other).

### Statistical methods

A descriptive analysis was made of the study population and the answers given. Qualitative variables were described using absolute frequencies and percentages, and quantitative variables using central trends, position, and dispersion by mean and standard deviation (SD). The social network was analyzed using exponential random graph models (ERGM) [33, 34] to assess how individual or shared characteristics influenced social links. ERGM are a class of exponential models that aims to predict the probability of a link between two nodes based on a set of individual and dyadic predictors of interest [35]. Sender and receiver characteristics were considered as individual predictors. Homophily for shared characteristics and mutuality (effect of reciprocal links) were considered as dyadic predictors. The baseline probability of a link is represented by an edges coefficient. Additional file 2: Table S1 contains a detailed description of all ERGM terms in our study. The analysis used R version 3.2.2 [36] and the Statnet library [37].

## Results

**Fig. 1:**
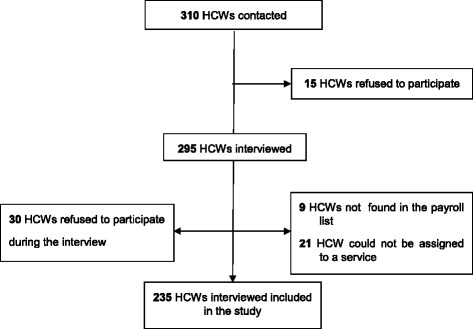
Flow chart

A total of 310 HCWs were contacted, representing 82.4% of the payroll list (*n* = 376). Of these (Fig. 1), 45 refused to participate (85.5% participation rate). There were no significant differences in demographic characteristics between the payroll list and the HCWs included (Table 1). The 235 HCWs included in the analysis generated 521 links, resulting in 466 valid links and 341 nodes (see Additional file 3). Each HCW interviewed cited a mean of 1.98 HCWs (SD 1.45, median 2, max 8) and received a mean of 1.36 citations (SD 1.5, median 1, max 7) from other HCWs. Cited HCWs who had not been interviewed received a mean of 1.39 citations (SD 0.85, median 1). Of HCWs interviewed, 38 cited no one, 88 were not cited by anyone else, and 21 were neither cited nor cited anyone. Of the 319 links between interviewed HCWs, 114 (35.74%) were mutual (the opposite link was present).

**Table 1:** Demographic characteristics of the payroll list and the participants included (interviewed, cited not interviewed, and total)

	Payroll list	Interviewed	Cited, not interviewed	Total included
Total (*n*)	376	235	106	341
Female (*n*, %)	284, 75.53%	185, 78.72%	83, 78.3%	268, 78.59%
Age (mean, SD)	42.84, 11.75	41.34, 11.47	42.32, 12.6	41.65, 11.82
Prof. category (*n*, %)				
Staff physician	67, 17.82%	40, 17.02%	20, 18.87%	60, 17.6%
Resident physician	40, 10.64%	19, 8.09%	16, 15.09%	35, 10.26%
Nurse	152, 40.43%	94, 40%	46, 43.4%	140, 41.06%
Auxiliary nurses/orderly	67, 17.82%	43, 18.3%	14, 13.21%	57, 16.72%
Administration/other	50, 13.3%	39, 16.6%	10, 9.43%	49, 14.37%
Department (*n*, %)				
A	209, 55.59%	131, 55.74%	40, 37.74%	171, 50.15%
B	167, 44.41%	104, 44.26%	33, 31.13%	137, 40.18%
Other			33, 31.13%	33, 9.68%
Vaccinated (*n*, %)	109, 28.99%	85, 36.17%	36, 33.96%	121, 35.48%
Positions of responsibility (*n*, %)	16, 4.26%	13, 5.53%	5, 4.72%	18, 5.28%

ERGM analysis of the network involved a number of different models. Table 2 shows the coefficients of the selected models. The odds ratios (ORs) of the probability of a link are obtained by exponentiation. The following ORs are taken from model 4. Homophily was significant for professional category (OR 3.13, 95% CI = 2.61–3.75), sex (OR 1.34, 95% CI = 1.09–1.62), age (OR 0.7, 95% CI = 0.63–0.78 per decade of difference), and department (OR 11.35, 95% CI = 8.17–15.64) but not for vaccination status (OR 1.02, 95% CI = 0.86–1.22), even in the simplistic model considering homophily by vaccination status alone (OR 0.94, 95% CI = 0.78–1.13). This means that, given two nodes, the OR of a link increased when the two nodes shared a category other than vaccination status. The sender effect was significant for staff physicians (OR 0.69, 95% CI 0.50–0.94) and nurses (OR 0.61, 95% CI 0.47–0.78), meaning that they were less likely to cite someone in the survey with respect to the “other” category. A vaccinated HCW was more likely to be named by their peers than a non-vaccinated HCW (OR 1.33, 95% CI 1.09–1.62) when all other factors were kept constant. Vaccinated HCWs were not significantly more likely to be sources of links (OR 1.12, 95% CI 0.92–1.37). The receiver effect of the variable age was significant (OR 1.26, 95% CI = 1.14–1.39 per extra decade of receiver HCW) as was the sender effect (OR 0.82, 95% CI 0.74–0.9 per extra decade of sender HCW). In all models used, mutuality had the largest effect and was always significant (OR 38.74, 95% CI 27.03–55.59). Fit diagnostics were checked for all the models included in Table 2 (see Additional file 4) and no indication of degeneracy was found.

**Table 2:** Coefficients of ERG models of the social network of two departments of HCWs around the 2010/2011 influenza vaccination campaign at HCB

	Model 1 (95% CI)	Model 2 (95% CI)	Model 3 (95% CI)	Model 4 (95% CI)
Edges	**−** *7.84* (**−**8.23, **−**7.45)	**−** *7.93* (**−**8.47, **−**7.39)	**−** *7.62* (**−**8.05, **−**7.18)	**−** *7.74* (**−**8.32, **−**7.16)
Homophily				
Professional category	*1.05* (0.87, 1.22)	*1.04* (0.86, 1.21)	*1.14* (0.96, 1.32)	*1.14* (0.96, 1.32)
Department	*2.41* (2.08, 2.73)	*2.42* (2.09, 2.74)	*2.43* (2.10, 2.75)	*2.43* (2.10, 2.75)
Sex	*0.27* (0.08, 0.47)	*0.28* (0.08, 0.48)	*0.29* (0.09, 0.50)	*0.29* (0.09, 0.49)
Vaccinated	0.02 (**−**0.15, 0.19)	0.02 (**−**0.15, 0.19)	0.02 (**−**0.15, 0.19)	0.02 (**−**0.15, 0.20)
Age (per extra decade of age difference between sender and receiver)	**−** *0.32* (**−**0.42, **−**0.22)	**−** *0.36* (**−**0.47, **−**0.26)	**−** *0.31* (**−**0.41, **−**0.21)	**−** *0.36* (**−**0.46, **−**0.25)
Receiver effects				
Vaccinated (y vs. n)	*0.31* (0.12, 0.51)	*0.30* (0.10, 0.49)	*0.29* (0.09, 0.48)	*0.28* (0.09, 0.48)
Positions of responsibility (*y* vs. *n*)		0.26 (**−**0.12, 0.63)	0.37 (**−**0.01, 0.74)	0.17 (**−**0.22, 0.55)
Age (per extra decade of receiver)		*0.22* (0.12, 0.31)		*0.23* (0.13, 0.33)
Sender effects				
Positions of responsibility (*y* vs. *n*)			*0.40* (0.01, 0.79)	*0.55* (0.15, 0.94)
Prof. category (reference “other”)				
Auxiliary nurses/orderly			**−**0.15 (**−**0.45, 0.14)	**−**0.15 (**−**0.45, 0.15)
Nurses			**−** *0.51* (**−**0.76, **−**0.26)	**−** *0.50* (**−**0.76, **−**0.25)
Staff physician			**−** *0.39* (**−**0.70, **−**0.08)	**−** *0.37* (**−**0.69, **−**0.06)
Resident phys.			**−**0.07 (**−**0.43, 0.28)	**−**0.14 (**−**0.51, 0.23)
Vaccinated (y vs. n)	0.16 (**−**0.04, 0.35)	0.17 (**−**0.02, 0.36)	0.10 (**−**0.10, 0.30)	0.12 (**−**0.08, 0.31)
Age (per extra decade of sender)		**−** *0.2* (**−**0.29, **−**0.1)		**−** *0.2* (**−**0.3, **−**0.1)
Mutuality	*3.62* (3.27, 3.98)	*3.68* (3.32, 4.04)	*3.59* (3.24, 3.95)	*3.66* (3.30, 4.02)
AIC	5049.00	5024.00	5033.00	5011.00
BIC	5136.00	5140.00	5178.00	5175.00

Italicization indicates statistical significance (*p* < 0.05)

*AIC* Akaike Information Criterion, *BIC* Bayesian Information Criterion

When asked to provide additional information about the reported relations, 86.48% of the 466 links were general comments about the campaign, 19.53% were joint participation in campaign activities, 8.8% reported having issued a recommendation (we did not register the sense), and 11.59% reported having received a recommendation. People stated they had known each other, on average, for 6.48 (SD 6.82) years. The frequency of meetings was daily (81.97%), weekly (15.67%), or monthly (2.36%). The topics usually discussed with the link were work (98.5%), personal affairs (79.4%), leisure activities (79.4%), and news (73.18%).

With respect to the second question on the perception of the vaccination status of their colleagues, 122 HCWs (51.91% of those interviewed) cited at least one other HCW they thought was vaccinated, and 94 of these (77.05%) cited only vaccinated HCWs. The positive predictive value (PPV) (percentage of cited HCWs who were actually vaccinated) was 86%.

## Discussion

We studied the influenza vaccination of HCWs and the social network defined by conversations around a vaccination campaign. A major finding is that similarity in vaccination behavior did not play a significant role in the probability of naming another HCW in our hospital. Links were more likely when individuals shared a professional category, sex, age, or department. In addition, some characteristics influenced participants citing more HCWs (being younger, having a position of responsibility, some professional categories) and others which increased the likelihood of being cited as a link (being vaccinated).

The lack of homophily according to vaccination behavior, also described for influenza vaccination in a friendship network of medical students [28], contrasts with other studies of advice networks in parental vaccination decisions [24–26] and in a contact network of influenza vaccination in high school students [27], where homophily by vaccination status was observed. The differences between our findings and those of other reports may be due to two reasons. First, different types of networks may yield different answers to the question of homophily by vaccination status. In contact networks, which are closely related to the spread of the virus, homophily by vaccination behavior may ultimately lead to the formation of unvaccinated clusters and thus reduce herd immunity [27, 38]. Advice-seeking, discussion (such as ours), and even friendship networks are probably more dependent on the cultural context, the type of vaccine or disease, and, in the case of HCWs, corporate culture. Both types of networks are interdependent, and, in the case of communicable diseases, social reinforcement of positive and negative opinions around vaccination [11] may increase the possibility of outbreaks through the spread of negative opinions about vaccination [39, 40]. Secondly, our study took into account homophily by other factors than those considered in other studies. In this respect, we would like to emphasize that the lack of homophily was observed even in the simplest model, which included only the homophilic effect by vaccination status. Homophily by professional category, age, or department has been reported in advice and friendship networks [18, 19]. Older and vaccinated HCWs were more frequently cited. This may indicate a pattern of professionals who are more often asked for advice or whose conversations are more frequently recalled. The seniority factor has also been observed in other studies [11, 12, 19].

The main limitation of the study is due to the cross-sectional design, which does not allow inferential conclusions on the impact of the campaigns to be drawn. The names of the two departments remain confidential but the results seem representative of the whole hospital since there were no significant differences in the study variables with the payroll list [31]. Services directly related to infectious diseases and infection control were avoided. In contrast with other behaviors or conditions, such as overweight and smoking, influenza vaccination could remain invisible to peers. Ideally, a question about the perception of the vaccination status of the HCWs named should have been included, but this could have compromised participation in the study. A separate question was thus added as a quality control, and a satisfactory PPV was obtained. A strength of the study is that it avoided sampling. The recruitment rate of 82.4% of the total payroll list, which included workers on leave and vacation and part-timers, is a good reflection of the entire workforce, given that interviewers repeatedly visited all wards and all shifts until no new HCW was found. Participation was also high. The reported mutuality of 35.7% is not high but is comparable to other studies of advice and discussion network [13, 16, 41, 42]. This rate is an indication that many conversations between two interviewed HCWs that actually occurred were only reported by one of them. A possible approach would be to treat this inconsistency as a reporting error and incorporate it into the model [41]. However, we decided not to symmetrize the network because we consider that the fact of recalling and reporting a conversation by a HCW may be of importance in their decision making, independently of whether it is also recalled by the target HCW. Caution should be taken about the generalization of the results on homophily, since they are closely related to the cultural and corporate contexts and we believe that the present study is an illustration of the relevance of SNA in the assessment and planning of health promotion campaigns aiming at peer-to-peer interaction.

This study is a first approach to the use of SNA as a tool in vaccination campaigns. From the perspective of health promotion, especially when aimed at participation, two situations that should be avoided may be detected by SNA. First, if health behaviors are grouped in closed communities, strategies to increase communication between peers can reinforce the beliefs of individuals in each group by reducing exposure to different points of view. Homophily due to factors other than health behavior may increase the exposure of individuals to diverse opinions and help changes in the behavior to spread more successfully [21]. Secondly, individuals opposed to the recommendations of the program may be more active in the network and have a higher probability of influencing others’ views, as has been reported in some online social networks [40]. Neither of these two possibilities was observed in our study. Since fostering participation has the risk that individuals issue messages that differ from the recommendations, SNA may be a useful tool for the assessment of the role played by a health behavior in a group and could shed light on the design, implementation, and evaluation of a health promotion campaign, which should be validated. In this social network, for example, messages could be tailored by professional category or strategies to foster communication among different professional categories could be implemented. Due to the lack of homophily by vaccination behavior, vaccine promotion actions aimed at fostering communication among HCWs should be continued. More research is needed to clarify situations in which the influenza vaccination status might be a homophilic factor in this kind of social network of HCWs.

## Conclusions

This study defines to whom HCWs relate in the context of a vaccination promotion campaign and shows that, in this social network, there is no homophily by vaccination status. Analysis of the social network of conversations around a health topic highlights the existing channels of communication and the relevance of the health behavior in the configuration of links.

## Additional files

Additional file 1: Survey. (DOCX 19 kb)
Additional file 2: Table S1. Interpretation of the coefficients of ERG models. (DOCX 12 kb)
Additional file 3: Study survey. (GRAPHML 63 kb)
Additional file 4: Fit diagnostics of ERGM models. (DOCX 775 kb)

## Abbreviations

AIC: Akaike Information Criterion
BIC: Bayesian Information Criterion
CI: Confidence interval
ERGM: Exponential random graph model
HCB: Hospital Clínic de Barcelona
HCW: Health care worker
HR: Human resources
OH: Occupational health
OR: Odds ratio
SD: Standard deviation
SNA: Social network analysis
