# Supplementary material for: The social network around influenza vaccination in health care workers: a cross-sectional study
Source: Implement Sci. 2016 Nov 24;11:152. doi: 10.1186/s13012-016-0522-3 (PMC5122207; doi:10.1186/s13012-016-0522-3)
Supplement: Additional file 1: — Survey. (DOCX 19 kb) [file 13012_2016_522_MOESM1_ESM.docx]

SURVEY

**Research Project: Social networks in the promotion of vaccination in health professionals**

We are interested in describing and drawing the network of healthcare professionals based on their relationship after the vaccination campaign. We are not interested in collecting your opinion on vaccination or what you decided to do this year but with whom you've spoken.

**In general, how is this used for?**

**- To determine the influence and scope of the social environment in the decision to be vaccinated**

**- To think about the diffusion of the messages of health promotion in the workplace.**

**What do we guarantee?**

**- We guarantee at all times the confidentiality of the statements of all employees. Data will be collected in a coded. Only the pollsters will know who has been interviewed.**

**- The data will be presented in a form that guarantees anonymity and ensures that individuals in a network cannot be recognized.**

**- We guarantee that no one will have access to individual answers, i.e., neither management nor supervisors will know who has responded at any time.**

**- The project has been approved by the Hospital Ethics Committee.**

*- Presentation of the study and verbal consent. Confidentiality is guaranteed.*

*- Check the inclusion criteria: that you are active and working in the selected service during the campaign.*

- This is an independent studio that is not concerned with your decision about vaccination.

**RESPONDENT CODE __ ___ ____ ___**

**Interviewer**

- **Interviewer 1**
- **Interviewer 2**
- **Interviewer 3**
- **Interviewer 4**

**Have you worked in this service during the last month (October)?**

- **Yes**
- **No**
- **A part of the last month. Which?____________**

**If no or only partly, why?**

- **Sick leave**
- **Worked in another hospital service. Which? ________**
- **Not working at the hospital.**

**Question 1. Who did you talk to or share some activity with with respect to the seasonal vaccination campaign?**

This includes: being vaccinated together, taking a photo together, talking to someone who has recommended vaccination or not or to whom you’ve recommended vaccination or not: always face-to-face activities either for or against vaccination. We do not wish to know the content of the relationship (we do not want to know what you think of them or what you think of vaccination or the campaign).

The number of persons you mention depends on you. Some people mention one or two colleagues, and some mention 10, while others mention no one. Feel free to name the people you want.

**Therefore, who did you talk to or share some activity with with respect to the seasonal vaccination campaign?**

*The interviewer firstly includes all the personal codes. Subsequently, when the spontaneous listing has been done, details will be collected.*

**RESPONDENT CODE __ ___ ____ ___**

| **Relationship**  **code** | **Type of relationship**  (Tick 1 or more)   - General comment about the campaign or vaccination - Activities (including vaccination) - Person who recommends vaccination/no vaccination (do not say which) - Person to whom you've recommended vaccination/no vaccination (do not say which) | **How long have you known them?**  Indicate in  __months or  __years | **How often do you speak with him/her?**   - Daily - Weekly - Monthly - Annually | **What do you usually talk about with this person** (tick 1 or more)?   - personal affairs - work - news, current affairs, sports - Leisure activities - Other____ |
| --- | --- | --- | --- | --- |
|  | (Tick 1 or more)   - General comment about the campaign or vaccination - Activities (including vaccination) - Person who recommends vaccination/no vaccination (do not say which) - Person to whom you've recommended vaccination/no vaccination (do not say which) | **How long have you known them?**  Indicate in  __months or  __years | **How often do you speak with him/her?**   - Daily - Weekly - Monthly - Annually | **What do you usually talk about with this person** (tick 1 or more)?   - personal affairs - work - news, current affairs, sports - Leisure activities - Other____ |
|  | (Tick 1 or more)   - General comment about the campaign or vaccination - Activities (including vaccination) - Person who recommends vaccination/no vaccination (do not say which) - Person to whom you've recommended vaccination/no vaccination (do not say which) | **How long have you known them?**  Indicate in  __months or  __years | **How often do you speak with him/her?**   - Daily - Weekly - Monthly - Annually | **What do you usually talk about with this person** (tick 1 or more)?   - personal affairs - work - news, current affairs, sports - Leisure activities - Other____ |

*Add as many copies of this page as necessary*

**RESPONDENT CODE __ ___ ____ ___**

**Question 2. Who do you know in hospital that has been vaccinated this season?**

The occupational risk prevention service already know who is vaccinated and who is not, and therefore we are not interested in knowing this but rather we wish to know who are the first persons who come to mind who you know have been vaccinated.

**So, who do you know who has been vaccinated this season?**

Please give a maximum of five names.

*Obtain the code of the worker assigned by the study.*

| **Code** |
| --- |
|  |
|  |
|  |
|  |

Thank you for your participation!

Preventive Medicine and Epidemiology Service

Occupational Risk Prevention Service

Department of Applied Mathematics, Polytechnic University of Catalonia
