# Supplementary material for: The social network around influenza vaccination in health care workers: a cross-sectional study
Source: Implement Sci. 2016 Nov 24;11:152. doi: 10.1186/s13012-016-0522-3 (PMC5122207; doi:10.1186/s13012-016-0522-3)
Supplement: Additional file 2: Table S1. — Interpretation of the coefficients of ERG models. (DOCX 12 kb) [file 13012_2016_522_MOESM2_ESM.docx]

| ERGM predictor | Interpretation of the coefficient |
| --- | --- |
| **Edges** | Baseline probability of a link X → Y between sender X and receiver Y |
| **Homophily** |  |
| Professional Category | Variation in the log-odds of a link X → Y when both sender X and receiver Y belong to the same professional category. Positive values favour homophily. |
| Institute | Variation in the log-odds of a link X → Y when both sender X and receiver Y belong to the same institute. Positive values favour homophily. |
| Sex | Variation in the log-odds of a link X → Y when both sender X and receiver Y have the same sex (both male or both female). Positive values favour homophily. |
| Vaccinated | Variation in the log-odds of a link X → Y when both sender X and receiver Y have the same vaccination status (either both vaccinated or both unvaccinated). Positive values favour homophily. |
| Age (per extra year of age difference between sender and receiver) | Variation in the log-odds of a link X → Y when the absolute value of the age of the receiver Y minus the age of the sender X increases by one year. Negative values favour homophily. |
| **Receiver Effects** |  |
| Vaccinated (y vs n) | Variation in the log-odds of a link X → Y when the receiver Y is vaccinated. Positive values indicate that vaccinated HCWs are more frequently cited. |
| Positions of  responsibility (y vs n) | Variation in the log-odds of a link X → Y when the receiver Y is in a position of responsibility. Positive values indicate that HCWs in a position of responsibility are more frequently cited. |
| Age (per extra year of receiver) | Variation in the log-odds of a link X → Y when the age of the receiver Y increases by one year. Positive values indicate that the older a HCW is the more frequently is cited. |
| **Sender Effects** |  |
| Positions of responsibility (y vs n) | Variation in the log-odds of a link X → Y when the sender X is in a position of responsibility. Positive values indicate that HCWs in a position of responsibility name more relationships. |
| Professional category  (reference “Other”) | Variation in the log-odds of a link X → Y when the professional category of the sender X is in one of the possible categories with respect to the “Other” category. Positive values indicate that HCWs in this category name more relationships than the “Other” category. |
| Vaccinated (y vs n) | Variation in the log-odds of a link X → Y when the sender X is vaccinated. Positive values indicate that vaccinated HCWs name more relationships. |
| Age (per extra year of sender) | Variation in the log-odds of a link X → Y when the age of the sender X increases by one year. Negative values indicate that the younger a HCW is the greater the number of relationships he or she cites. |
| **Mutuality** | Variation in the log-odds of a link X → Y when the edge Y → X is present. |

**Table S1:** Interpretation of the coefficients of ERG models.
