# Supplementary material for: The social network around influenza vaccination in health care workers: a cross-sectional study
Source: Implement Sci. 2016 Nov 24;11:152. doi: 10.1186/s13012-016-0522-3 (PMC5122207; doi:10.1186/s13012-016-0522-3)
Supplement: Additional file 4: — Fit diagnostics of ERGM models. (DOCX 775 kb) [file 13012_2016_522_MOESM4_ESM.docx]

Fit Diagnostics of ERGM models

Supplementary Material to the paper

*The Social Network around Influenza Vaccination in Health Care Workers: a Cross-Sectional Study*

In this supplementary material we present the fit diagnostics obtained by the 'mcmc.diagnostics()' routine from the STATNET suite (see paper for details) to the four ERGM models considered in Table 2.

Model 1

## Sample statistics summary:
##
## Iterations = 65536:67170304
## Thinning interval = 4096
## Number of chains = 1
## Sample size per chain = 16384
##
## 1. Empirical mean and standard deviation for each variable,
## plus standard error of the mean:
##
## Mean SD Naive SE Time-series SE
## edges 0.6673 23.881 0.18657 0.6087
## nodeifactor.Vaccinated.TRUE 0.5455 14.922 0.11658 0.3868
## nodeofactor.Vaccinated.TRUE 0.2042 14.455 0.11293 0.3882
## nodematch.ProfCat 1.1636 18.228 0.14241 0.5648
## nodematch.Unit 0.5572 23.010 0.17977 0.6136
## nodematch.Sex 0.0603 20.836 0.16278 0.5546
## nodematch.Vaccinated 1.4255 17.381 0.13579 0.4416
## absdiff.Age 1.7067 30.349 0.23710 0.6098
## mutual 0.1556 7.675 0.05996 0.3043
##
## 2. Quantiles for each variable:
##
## 2.5% 25% 50% 75% 97.5%
## edges -46.0 -16.0 1.0 17.0 48.0
## nodeifactor.Vaccinated.TRUE -28.0 -10.0 0.0 11.0 30.0
## nodeofactor.Vaccinated.TRUE -28.0 -10.0 0.0 10.0 29.0
## nodematch.ProfCat -34.0 -11.0 1.0 13.0 37.0
## nodematch.Unit -44.0 -15.0 1.0 16.0 46.0
## nodematch.Sex -40.0 -14.0 0.0 14.0 41.0
## nodematch.Vaccinated -32.0 -10.0 1.0 13.0 36.0
## absdiff.Age -57.4 -18.6 1.5 21.7 62.6
## mutual -15.0 -5.0 0.0 5.0 16.0
##
##
## Sample statistics cross-correlations:
## edges nodeifactor.Vaccinated.TRUE
## edges 1.0000000 0.6958762
## nodeifactor.Vaccinated.TRUE 0.6958762 1.0000000
## nodeofactor.Vaccinated.TRUE 0.6844522 0.6281930
## nodematch.ProfCat 0.7712121 0.5254011
## nodematch.Unit 0.9624815 0.6746441
## nodematch.Sex 0.8781220 0.6136757
## nodematch.Vaccinated 0.7322750 0.4045932
## absdiff.Age 0.7580204 0.5216639
## mutual 0.6225487 0.4691157
## nodeofactor.Vaccinated.TRUE nodematch.ProfCat
## edges 0.6844522 0.7712121
## nodeifactor.Vaccinated.TRUE 0.6281930 0.5254011
## nodeofactor.Vaccinated.TRUE 1.0000000 0.5207500
## nodematch.ProfCat 0.5207500 1.0000000
## nodematch.Unit 0.6617840 0.7484690
## nodematch.Sex 0.6101007 0.7186735
## nodematch.Vaccinated 0.4171782 0.5816714
## absdiff.Age 0.5065658 0.5576384
## mutual 0.4827253 0.6031400
## nodematch.Unit nodematch.Sex
## edges 0.9624815 0.8781220
## nodeifactor.Vaccinated.TRUE 0.6746441 0.6136757
## nodeofactor.Vaccinated.TRUE 0.6617840 0.6101007
## nodematch.ProfCat 0.7484690 0.7186735
## nodematch.Unit 1.0000000 0.8471826
## nodematch.Sex 0.8471826 1.0000000
## nodematch.Vaccinated 0.7046961 0.6407214
## absdiff.Age 0.7222867 0.6680745
## mutual 0.6418909 0.5809634
## nodematch.Vaccinated absdiff.Age mutual
## edges 0.7322750 0.7580204 0.6225487
## nodeifactor.Vaccinated.TRUE 0.4045932 0.5216639 0.4691157
## nodeofactor.Vaccinated.TRUE 0.4171782 0.5065658 0.4827253
## nodematch.ProfCat 0.5816714 0.5576384 0.6031400
## nodematch.Unit 0.7046961 0.7222867 0.6418909
## nodematch.Sex 0.6407214 0.6680745 0.5809634
## nodematch.Vaccinated 1.0000000 0.5535246 0.4632382
## absdiff.Age 0.5535246 1.0000000 0.4053311
## mutual 0.4632382 0.4053311 1.0000000
##
## Sample statistics auto-correlation:
## Chain 1
## edges nodeifactor.Vaccinated.TRUE
## Lag 0 1.0000000 1.0000000
## Lag 4096 0.4464432 0.4456518
## Lag 8192 0.3591563 0.3556038
## Lag 12288 0.3290071 0.3147510
## Lag 16384 0.2930482 0.2883071
## Lag 20480 0.2678746 0.2653712
## nodeofactor.Vaccinated.TRUE nodematch.ProfCat nodematch.Unit
## Lag 0 1.0000000 1.0000000 1.0000000
## Lag 4096 0.4499925 0.5910036 0.4836540
## Lag 8192 0.3651262 0.4874147 0.3911231
## Lag 12288 0.3247427 0.4407551 0.3605845
## Lag 16384 0.2983874 0.4071446 0.3198491
## Lag 20480 0.2773940 0.3784601 0.2924890
## nodematch.Sex nodematch.Vaccinated absdiff.Age mutual
## Lag 0 1.0000000 1.0000000 1.0000000 1.0000000
## Lag 4096 0.4790311 0.4494971 0.3165312 0.9084367
## Lag 8192 0.3888952 0.3662570 0.2435307 0.8316170
## Lag 12288 0.3512424 0.3331299 0.2210827 0.7670107
## Lag 16384 0.3164991 0.2943350 0.1880320 0.7089306
## Lag 20480 0.2912256 0.2657961 0.1774221 0.6536976
##
## Sample statistics burn-in diagnostic (Geweke):
## Chain 1
##
## Fraction in 1st window = 0.1
## Fraction in 2nd window = 0.5
##
## edges nodeifactor.Vaccinated.TRUE
## -0.2907 -0.6387
## nodeofactor.Vaccinated.TRUE nodematch.ProfCat
## -1.1020 -0.7083
## nodematch.Unit nodematch.Sex
## -0.2732 -0.9457
## nodematch.Vaccinated absdiff.Age
## 0.7516 -1.2524
## mutual
## -0.6207
##
## Individual P-values (lower = worse):
## edges nodeifactor.Vaccinated.TRUE
## 0.7712632 0.5230459
## nodeofactor.Vaccinated.TRUE nodematch.ProfCat
## 0.2704724 0.4787439
## nodematch.Unit nodematch.Sex
## 0.7847330 0.3443085
## nodematch.Vaccinated absdiff.Age
## 0.4523117 0.2104420
## mutual
## 0.5348037
## Joint P-value (lower = worse): 0.2510962 .

## Loading required namespace: latticeExtra


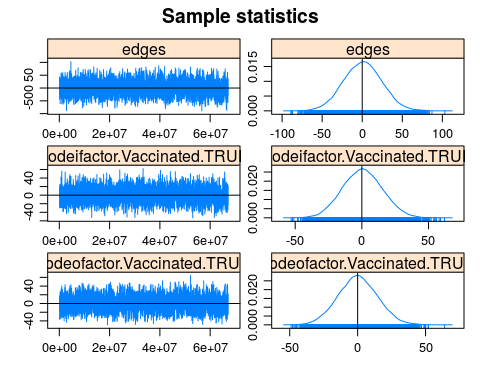

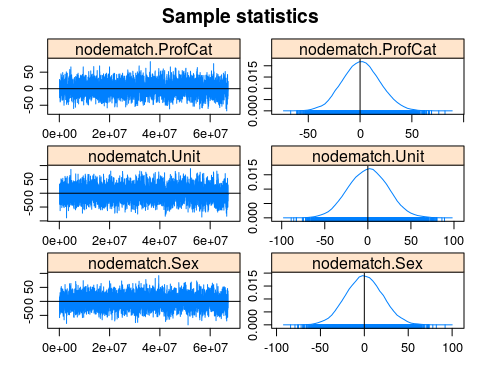

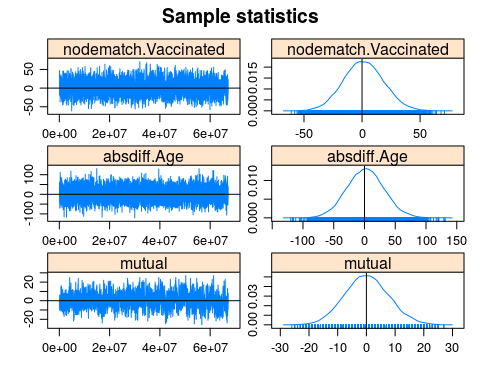


##
## MCMC diagnostics shown here are from the last round of simulation, prior to computation of final parameter estimates. Because the final estimates are refinements of those used for this simulation run, these diagnostics may understate model performance. To directly assess the performance of the final model on in-model statistics, please use the GOF command: gof(ergmFitObject, GOF=~model).

Model 2

## Sample statistics summary:
##
## Iterations = 65536:67170304
## Thinning interval = 4096
## Number of chains = 1
## Sample size per chain = 16384
##
## 1. Empirical mean and standard deviation for each variable,
## plus standard error of the mean:
##
## Mean SD Naive SE Time-series SE
## edges 2.3599 23.589 0.18429 0.5638
## nodeifactor.Vaccinated.TRUE 1.0406 14.486 0.11317 0.3659
## nodeofactor.Vaccinated.TRUE 1.6193 14.040 0.10968 0.3615
## nodematch.ProfCat 1.9623 17.943 0.14018 0.5291
## nodematch.Unit 2.2133 22.642 0.17689 0.5603
## nodeifactor.Responsibility.TRUE 0.2831 5.801 0.04532 0.1336
## nodematch.Sex 1.9315 20.833 0.16276 0.5362
## nodematch.Vaccinated 1.3440 17.030 0.13305 0.4476
## absdiff.Age 0.8695 29.796 0.23278 0.5567
## nodeicov.Age 9.7966 104.957 0.81997 2.4821
## nodeocov.Age 10.2732 99.811 0.77977 2.4586
## mutual 0.8469 7.341 0.05735 0.2729
##
## 2. Quantiles for each variable:
##
## 2.5% 25% 50% 75% 97.5%
## edges -43.0 -14.00 2.0 18.0 49.00
## nodeifactor.Vaccinated.TRUE -27.0 -9.00 1.0 11.0 30.00
## nodeofactor.Vaccinated.TRUE -25.0 -8.00 1.0 11.0 30.00
## nodematch.ProfCat -33.0 -10.00 2.0 14.0 38.00
## nodematch.Unit -42.0 -13.00 2.0 17.0 47.00
## nodeifactor.Responsibility.TRUE -11.0 -4.00 0.0 4.0 12.00
## nodematch.Sex -38.0 -12.00 2.0 16.0 44.00
## nodematch.Vaccinated -32.0 -10.00 1.0 13.0 36.00
## absdiff.Age -56.9 -19.50 0.5 20.8 59.74
## nodeicov.Age -194.5 -61.22 9.3 79.8 216.64
## nodeocov.Age -183.1 -57.80 9.7 76.9 208.20
## mutual -13.0 -4.00 1.0 6.0 16.00
##
##
## Sample statistics cross-correlations:
## edges nodeifactor.Vaccinated.TRUE
## edges 1.0000000 0.6801984
## nodeifactor.Vaccinated.TRUE 0.6801984 1.0000000
## nodeofactor.Vaccinated.TRUE 0.6631267 0.5883755
## nodematch.ProfCat 0.7671579 0.5103960
## nodematch.Unit 0.9618004 0.6524633
## nodeifactor.Responsibility.TRUE 0.2652694 0.2156377
## nodematch.Sex 0.8756906 0.5995524
## nodematch.Vaccinated 0.7262461 0.3845764
## absdiff.Age 0.7527366 0.5133060
## nodeicov.Age 0.9698744 0.6599329
## nodeocov.Age 0.9672374 0.6559232
## mutual 0.6111022 0.4262424
## nodeofactor.Vaccinated.TRUE
## edges 0.6631267
## nodeifactor.Vaccinated.TRUE 0.5883755
## nodeofactor.Vaccinated.TRUE 1.0000000
## nodematch.ProfCat 0.4978462
## nodematch.Unit 0.6380948
## nodeifactor.Responsibility.TRUE 0.1815198
## nodematch.Sex 0.5835664
## nodematch.Vaccinated 0.4041081
## absdiff.Age 0.4960665
## nodeicov.Age 0.6414025
## nodeocov.Age 0.6433582
## mutual 0.4372496
## nodematch.ProfCat nodematch.Unit
## edges 0.7671579 0.9618004
## nodeifactor.Vaccinated.TRUE 0.5103960 0.6524633
## nodeofactor.Vaccinated.TRUE 0.4978462 0.6380948
## nodematch.ProfCat 1.0000000 0.7428848
## nodematch.Unit 0.7428848 1.0000000
## nodeifactor.Responsibility.TRUE 0.2390466 0.2447207
## nodematch.Sex 0.7048168 0.8436723
## nodematch.Vaccinated 0.5622367 0.6994394
## absdiff.Age 0.5583201 0.7179245
## nodeicov.Age 0.7525366 0.9314120
## nodeocov.Age 0.7594456 0.9311989
## mutual 0.5874535 0.6269297
## nodeifactor.Responsibility.TRUE
## edges 0.2652694
## nodeifactor.Vaccinated.TRUE 0.2156377
## nodeofactor.Vaccinated.TRUE 0.1815198
## nodematch.ProfCat 0.2390466
## nodematch.Unit 0.2447207
## nodeifactor.Responsibility.TRUE 1.0000000
## nodematch.Sex 0.2080304
## nodematch.Vaccinated 0.1800299
## absdiff.Age 0.2487454
## nodeicov.Age 0.3206680
## nodeocov.Age 0.2884061
## mutual 0.1360177
## nodematch.Sex nodematch.Vaccinated
## edges 0.8756906 0.7262461
## nodeifactor.Vaccinated.TRUE 0.5995524 0.3845764
## nodeofactor.Vaccinated.TRUE 0.5835664 0.4041081
## nodematch.ProfCat 0.7048168 0.5622367
## nodematch.Unit 0.8436723 0.6994394
## nodeifactor.Responsibility.TRUE 0.2080304 0.1800299
## nodematch.Sex 1.0000000 0.6372513
## nodematch.Vaccinated 0.6372513 1.0000000
## absdiff.Age 0.6516001 0.5538925
## nodeicov.Age 0.8537253 0.7072573
## nodeocov.Age 0.8552817 0.7038489
## mutual 0.5651934 0.4356455
## absdiff.Age nodeicov.Age nodeocov.Age
## edges 0.7527366 0.9698744 0.9672374
## nodeifactor.Vaccinated.TRUE 0.5133060 0.6599329 0.6559232
## nodeofactor.Vaccinated.TRUE 0.4960665 0.6414025 0.6433582
## nodematch.ProfCat 0.5583201 0.7525366 0.7594456
## nodematch.Unit 0.7179245 0.9314120 0.9311989
## nodeifactor.Responsibility.TRUE 0.2487454 0.3206680 0.2884061
## nodematch.Sex 0.6516001 0.8537253 0.8552817
## nodematch.Vaccinated 0.5538925 0.7072573 0.7038489
## absdiff.Age 1.0000000 0.7629767 0.7093365
## nodeicov.Age 0.7629767 1.0000000 0.9701556
## nodeocov.Age 0.7093365 0.9701556 1.0000000
## mutual 0.3812464 0.5789611 0.6121001
## mutual
## edges 0.6111022
## nodeifactor.Vaccinated.TRUE 0.4262424
## nodeofactor.Vaccinated.TRUE 0.4372496
## nodematch.ProfCat 0.5874535
## nodematch.Unit 0.6269297
## nodeifactor.Responsibility.TRUE 0.1360177
## nodematch.Sex 0.5651934
## nodematch.Vaccinated 0.4356455
## absdiff.Age 0.3812464
## nodeicov.Age 0.5789611
## nodeocov.Age 0.6121001
## mutual 1.0000000
##
## Sample statistics auto-correlation:
## Chain 1
## edges nodeifactor.Vaccinated.TRUE
## Lag 0 1.0000000 1.0000000
## Lag 4096 0.4435106 0.4131563
## Lag 8192 0.3505805 0.3129144
## Lag 12288 0.3008851 0.2648949
## Lag 16384 0.2685842 0.2421735
## Lag 20480 0.2494350 0.2073620
## nodeofactor.Vaccinated.TRUE nodematch.ProfCat nodematch.Unit
## Lag 0 1.0000000 1.0000000 1.0000000
## Lag 4096 0.4256364 0.5846433 0.4746850
## Lag 8192 0.3236009 0.4683921 0.3732284
## Lag 12288 0.2833732 0.4109201 0.3205724
## Lag 16384 0.2508575 0.3791373 0.2879435
## Lag 20480 0.2372101 0.3504130 0.2699004
## nodeifactor.Responsibility.TRUE nodematch.Sex
## Lag 0 1.0000000 1.0000000
## Lag 4096 0.4070007 0.4735191
## Lag 8192 0.2844491 0.3805555
## Lag 12288 0.2363644 0.3318763
## Lag 16384 0.2031422 0.2978449
## Lag 20480 0.1800480 0.2774906
## nodematch.Vaccinated absdiff.Age nodeicov.Age nodeocov.Age
## Lag 0 1.0000000 1.0000000 1.0000000 1.0000000
## Lag 4096 0.4325967 0.3180722 0.4324322 0.4610185
## Lag 8192 0.3408876 0.2307004 0.3382895 0.3715493
## Lag 12288 0.3022836 0.1935471 0.2892406 0.3166250
## Lag 16384 0.2663166 0.1622991 0.2555715 0.2889550
## Lag 20480 0.2432193 0.1477140 0.2396161 0.2637887
## mutual
## Lag 0 1.0000000
## Lag 4096 0.8990310
## Lag 8192 0.8161088
## Lag 12288 0.7425635
## Lag 16384 0.6798165
## Lag 20480 0.6253506
##
## Sample statistics burn-in diagnostic (Geweke):
## Chain 1
##
## Fraction in 1st window = 0.1
## Fraction in 2nd window = 0.5
##
## edges nodeifactor.Vaccinated.TRUE
## -2.15091 -2.69919
## nodeofactor.Vaccinated.TRUE nodematch.ProfCat
## -3.05699 -2.18725
## nodematch.Unit nodeifactor.Responsibility.TRUE
## -2.18746 -0.08493
## nodematch.Sex nodematch.Vaccinated
## -1.29506 -1.00967
## absdiff.Age nodeicov.Age
## -2.26306 -1.57951
## nodeocov.Age mutual
## -1.65154 -1.54724
##
## Individual P-values (lower = worse):
## edges nodeifactor.Vaccinated.TRUE
## 0.031483253 0.006950819
## nodeofactor.Vaccinated.TRUE nodematch.ProfCat
## 0.002235736 0.028724477
## nodematch.Unit nodeifactor.Responsibility.TRUE
## 0.028708859 0.932315096
## nodematch.Sex nodematch.Vaccinated
## 0.195299131 0.312655272
## absdiff.Age nodeicov.Age
## 0.023632068 0.114220207
## nodeocov.Age mutual
## 0.098628612 0.121804265
## Joint P-value (lower = worse): 0.03546549 .


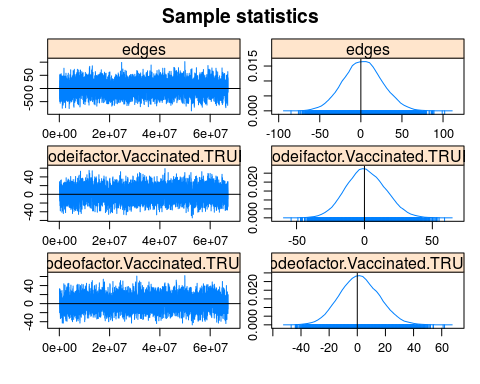

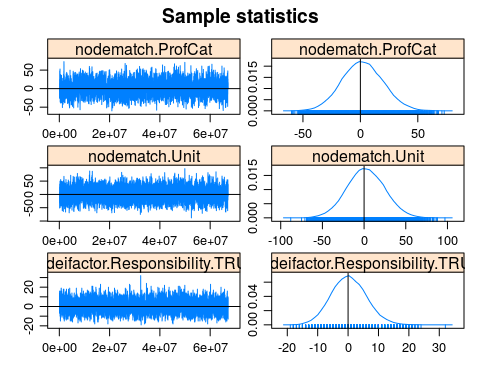

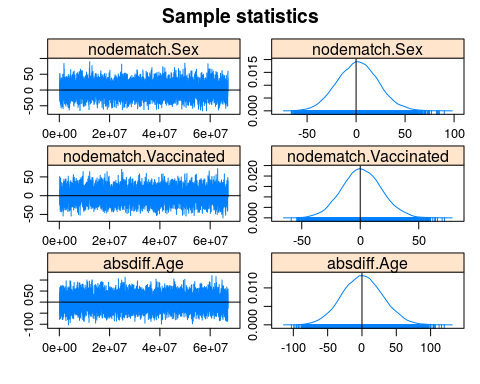

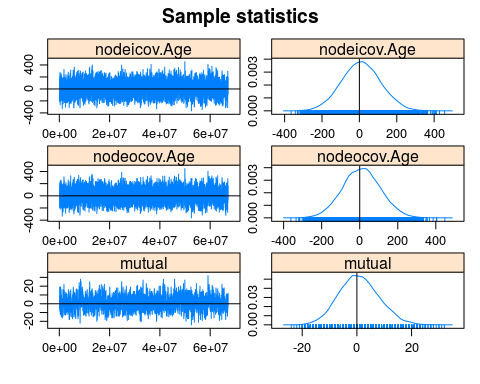


##
## MCMC diagnostics shown here are from the last round of simulation, prior to computation of final parameter estimates. Because the final estimates are refinements of those used for this simulation run, these diagnostics may understate model performance. To directly assess the performance of the final model on in-model statistics, please use the GOF command: gof(ergmFitObject, GOF=~model).

Model 3

## Sample statistics summary:
##
## Iterations = 65536:67170304
## Thinning interval = 4096
## Number of chains = 1
## Sample size per chain = 16384
##
## 1. Empirical mean and standard deviation for each variable,
## plus standard error of the mean:
##
## Mean SD Naive SE Time-series SE
## edges 1.88269 24.051 0.18789 0.6717
## nodeifactor.Vaccinated.TRUE 0.16730 14.612 0.11416 0.4057
## nodeofactor.Vaccinated.TRUE -0.33386 14.127 0.11037 0.3846
## nodematch.ProfCat 2.33997 18.885 0.14754 0.6566
## nodematch.Unit 1.79279 23.165 0.18098 0.6712
## nodematch.Sex 1.66113 21.229 0.16585 0.5970
## nodematch.Vaccinated 1.35199 17.296 0.13513 0.4263
## absdiff.Age 1.34900 30.385 0.23738 0.6279
## mutual 0.95032 7.664 0.05987 0.3125
## nodeifactor.Responsibility.TRUE -0.28979 5.749 0.04492 0.1852
## nodeofactor.Responsibility.TRUE -0.00885 5.635 0.04403 0.1646
## nodeofactor.ProfCat.Auxiliary 0.66168 9.592 0.07493 0.2496
## nodeofactor.ProfCat.Nurse 0.06573 15.647 0.12224 0.4331
## nodeofactor.ProfCat.Resident Phys. 0.47766 6.925 0.05410 0.2207
## nodeofactor.ProfCat.Staff Phys. -0.33954 8.461 0.06610 0.1947
##
## 2. Quantiles for each variable:
##
## 2.5% 25% 50% 75% 97.5%
## edges -45.0 -14.0 2.0 18.0 49.00
## nodeifactor.Vaccinated.TRUE -28.0 -10.0 0.0 10.0 29.00
## nodeofactor.Vaccinated.TRUE -28.0 -10.0 0.0 9.0 28.00
## nodematch.ProfCat -34.0 -11.0 2.0 15.0 40.00
## nodematch.Unit -43.0 -14.0 2.0 18.0 47.00
## nodematch.Sex -40.0 -13.0 2.0 16.0 43.00
## nodematch.Vaccinated -32.0 -10.0 1.0 13.0 36.00
## absdiff.Age -57.2 -19.4 0.6 21.6 63.24
## mutual -14.0 -4.0 1.0 6.0 17.00
## nodeifactor.Responsibility.TRUE -11.0 -4.0 0.0 3.0 11.00
## nodeofactor.Responsibility.TRUE -11.0 -4.0 0.0 4.0 12.00
## nodeofactor.ProfCat.Auxiliary -18.0 -6.0 1.0 7.0 20.00
## nodeofactor.ProfCat.Nurse -30.0 -10.0 0.0 10.0 32.00
## nodeofactor.ProfCat.Resident Phys. -13.0 -4.0 0.0 5.0 14.00
## nodeofactor.ProfCat.Staff Phys. -16.0 -6.0 -1.0 5.0 17.00
##
##
## Sample statistics cross-correlations:
## edges nodeifactor.Vaccinated.TRUE
## edges 1.0000000 0.6906267
## nodeifactor.Vaccinated.TRUE 0.6906267 1.0000000
## nodeofactor.Vaccinated.TRUE 0.6674588 0.5903133
## nodematch.ProfCat 0.7811639 0.5290619
## nodematch.Unit 0.9638858 0.6648153
## nodematch.Sex 0.8828660 0.6084373
## nodematch.Vaccinated 0.7310608 0.3951294
## absdiff.Age 0.7601803 0.5214507
## mutual 0.6316494 0.4525158
## nodeifactor.Responsibility.TRUE 0.2913688 0.2313943
## nodeofactor.Responsibility.TRUE 0.2910758 0.2011194
## nodeofactor.ProfCat.Auxiliary 0.4066747 0.3221898
## nodeofactor.ProfCat.Nurse 0.6827254 0.4326083
## nodeofactor.ProfCat.Resident Phys. 0.3110432 0.2343815
## nodeofactor.ProfCat.Staff Phys. 0.3958012 0.2718504
## nodeofactor.Vaccinated.TRUE
## edges 0.6674588
## nodeifactor.Vaccinated.TRUE 0.5903133
## nodeofactor.Vaccinated.TRUE 1.0000000
## nodematch.ProfCat 0.5188587
## nodematch.Unit 0.6450887
## nodematch.Sex 0.5902742
## nodematch.Vaccinated 0.4097172
## absdiff.Age 0.4976171
## mutual 0.4624682
## nodeifactor.Responsibility.TRUE 0.2021210
## nodeofactor.Responsibility.TRUE 0.2235303
## nodeofactor.ProfCat.Auxiliary 0.3573616
## nodeofactor.ProfCat.Nurse 0.3956578
## nodeofactor.ProfCat.Resident Phys. 0.2580225
## nodeofactor.ProfCat.Staff Phys. 0.2388098
## nodematch.ProfCat nodematch.Unit
## edges 0.7811639 0.9638858
## nodeifactor.Vaccinated.TRUE 0.5290619 0.6648153
## nodeofactor.Vaccinated.TRUE 0.5188587 0.6450887
## nodematch.ProfCat 1.0000000 0.7588826
## nodematch.Unit 0.7588826 1.0000000
## nodematch.Sex 0.7227278 0.8536655
## nodematch.Vaccinated 0.5676021 0.7045722
## absdiff.Age 0.5628012 0.7262960
## mutual 0.6229710 0.6487577
## nodeifactor.Responsibility.TRUE 0.2584576 0.2765357
## nodeofactor.Responsibility.TRUE 0.2672755 0.2767145
## nodeofactor.ProfCat.Auxiliary 0.2595314 0.3939489
## nodeofactor.ProfCat.Nurse 0.6635877 0.6583554
## nodeofactor.ProfCat.Resident Phys. 0.1740960 0.2995791
## nodeofactor.ProfCat.Staff Phys. 0.2786634 0.3774638
## nodematch.Sex nodematch.Vaccinated
## edges 0.8828660 0.7310608
## nodeifactor.Vaccinated.TRUE 0.6084373 0.3951294
## nodeofactor.Vaccinated.TRUE 0.5902742 0.4097172
## nodematch.ProfCat 0.7227278 0.5676021
## nodematch.Unit 0.8536655 0.7045722
## nodematch.Sex 1.0000000 0.6349986
## nodematch.Vaccinated 0.6349986 1.0000000
## absdiff.Age 0.6689205 0.5571674
## mutual 0.5937606 0.4439846
## nodeifactor.Responsibility.TRUE 0.2308713 0.2045304
## nodeofactor.Responsibility.TRUE 0.2247298 0.2039671
## nodeofactor.ProfCat.Auxiliary 0.4127485 0.3020187
## nodeofactor.ProfCat.Nurse 0.6355178 0.5074438
## nodeofactor.ProfCat.Resident Phys. 0.1917132 0.2256117
## nodeofactor.ProfCat.Staff Phys. 0.2837983 0.2911682
## absdiff.Age mutual
## edges 0.7601803 0.6316494
## nodeifactor.Vaccinated.TRUE 0.5214507 0.4525158
## nodeofactor.Vaccinated.TRUE 0.4976171 0.4624682
## nodematch.ProfCat 0.5628012 0.6229710
## nodematch.Unit 0.7262960 0.6487577
## nodematch.Sex 0.6689205 0.5937606
## nodematch.Vaccinated 0.5571674 0.4439846
## absdiff.Age 1.0000000 0.4160517
## mutual 0.4160517 1.0000000
## nodeifactor.Responsibility.TRUE 0.2605765 0.2154983
## nodeofactor.Responsibility.TRUE 0.2491020 0.2143855
## nodeofactor.ProfCat.Auxiliary 0.3253789 0.2352502
## nodeofactor.ProfCat.Nurse 0.5291773 0.4475672
## nodeofactor.ProfCat.Resident Phys. 0.1811706 0.2082233
## nodeofactor.ProfCat.Staff Phys. 0.3052912 0.2402174
## nodeifactor.Responsibility.TRUE
## edges 0.29136879
## nodeifactor.Vaccinated.TRUE 0.23139432
## nodeofactor.Vaccinated.TRUE 0.20212098
## nodematch.ProfCat 0.25845762
## nodematch.Unit 0.27653569
## nodematch.Sex 0.23087126
## nodematch.Vaccinated 0.20453038
## absdiff.Age 0.26057652
## mutual 0.21549834
## nodeifactor.Responsibility.TRUE 1.00000000
## nodeofactor.Responsibility.TRUE 0.35308391
## nodeofactor.ProfCat.Auxiliary 0.04085193
## nodeofactor.ProfCat.Nurse 0.27728476
## nodeofactor.ProfCat.Resident Phys. 0.03195475
## nodeofactor.ProfCat.Staff Phys. 0.12343237
## nodeofactor.Responsibility.TRUE
## edges 0.291075837
## nodeifactor.Vaccinated.TRUE 0.201119422
## nodeofactor.Vaccinated.TRUE 0.223530309
## nodematch.ProfCat 0.267275529
## nodematch.Unit 0.276714471
## nodematch.Sex 0.224729815
## nodematch.Vaccinated 0.203967096
## absdiff.Age 0.249102034
## mutual 0.214385543
## nodeifactor.Responsibility.TRUE 0.353083906
## nodeofactor.Responsibility.TRUE 1.000000000
## nodeofactor.ProfCat.Auxiliary -0.014523148
## nodeofactor.ProfCat.Nurse 0.319679887
## nodeofactor.ProfCat.Resident Phys. -0.001324286
## nodeofactor.ProfCat.Staff Phys. 0.144934697
## nodeofactor.ProfCat.Auxiliary
## edges 0.40667470
## nodeifactor.Vaccinated.TRUE 0.32218979
## nodeofactor.Vaccinated.TRUE 0.35736157
## nodematch.ProfCat 0.25953138
## nodematch.Unit 0.39394890
## nodematch.Sex 0.41274847
## nodematch.Vaccinated 0.30201870
## absdiff.Age 0.32537890
## mutual 0.23525021
## nodeifactor.Responsibility.TRUE 0.04085193
## nodeofactor.Responsibility.TRUE -0.01452315
## nodeofactor.ProfCat.Auxiliary 1.00000000
## nodeofactor.ProfCat.Nurse 0.01268720
## nodeofactor.ProfCat.Resident Phys. -0.01372882
## nodeofactor.ProfCat.Staff Phys. 0.01409757
## nodeofactor.ProfCat.Nurse
## edges 0.68272537
## nodeifactor.Vaccinated.TRUE 0.43260826
## nodeofactor.Vaccinated.TRUE 0.39565776
## nodematch.ProfCat 0.66358771
## nodematch.Unit 0.65835544
## nodematch.Sex 0.63551782
## nodematch.Vaccinated 0.50744379
## absdiff.Age 0.52917732
## mutual 0.44756719
## nodeifactor.Responsibility.TRUE 0.27728476
## nodeofactor.Responsibility.TRUE 0.31967989
## nodeofactor.ProfCat.Auxiliary 0.01268720
## nodeofactor.ProfCat.Nurse 1.00000000
## nodeofactor.ProfCat.Resident Phys. 0.02632827
## nodeofactor.ProfCat.Staff Phys. 0.04230712
## nodeofactor.ProfCat.Resident Phys.
## edges 0.311043249
## nodeifactor.Vaccinated.TRUE 0.234381461
## nodeofactor.Vaccinated.TRUE 0.258022507
## nodematch.ProfCat 0.174096034
## nodematch.Unit 0.299579083
## nodematch.Sex 0.191713159
## nodematch.Vaccinated 0.225611651
## absdiff.Age 0.181170630
## mutual 0.208223258
## nodeifactor.Responsibility.TRUE 0.031954753
## nodeofactor.Responsibility.TRUE -0.001324286
## nodeofactor.ProfCat.Auxiliary -0.013728820
## nodeofactor.ProfCat.Nurse 0.026328272
## nodeofactor.ProfCat.Resident Phys. 1.000000000
## nodeofactor.ProfCat.Staff Phys. 0.032762599
## nodeofactor.ProfCat.Staff Phys.
## edges 0.39580123
## nodeifactor.Vaccinated.TRUE 0.27185036
## nodeofactor.Vaccinated.TRUE 0.23880981
## nodematch.ProfCat 0.27866341
## nodematch.Unit 0.37746382
## nodematch.Sex 0.28379828
## nodematch.Vaccinated 0.29116824
## absdiff.Age 0.30529124
## mutual 0.24021745
## nodeifactor.Responsibility.TRUE 0.12343237
## nodeofactor.Responsibility.TRUE 0.14493470
## nodeofactor.ProfCat.Auxiliary 0.01409757
## nodeofactor.ProfCat.Nurse 0.04230712
## nodeofactor.ProfCat.Resident Phys. 0.03276260
## nodeofactor.ProfCat.Staff Phys. 1.00000000
##
## Sample statistics auto-correlation:
## Chain 1
## edges nodeifactor.Vaccinated.TRUE
## Lag 0 1.0000000 1.0000000
## Lag 4096 0.4649488 0.4265336
## Lag 8192 0.3670664 0.3079628
## Lag 12288 0.3292482 0.2665541
## Lag 16384 0.2987479 0.2405078
## Lag 20480 0.2737476 0.2205897
## nodeofactor.Vaccinated.TRUE nodematch.ProfCat nodematch.Unit
## Lag 0 1.0000000 1.0000000 1.0000000
## Lag 4096 0.4281315 0.6163486 0.4993405
## Lag 8192 0.3285702 0.5057090 0.3964537
## Lag 12288 0.2967283 0.4567661 0.3569497
## Lag 16384 0.2634472 0.4186201 0.3236847
## Lag 20480 0.2407380 0.3929551 0.2975468
## nodematch.Sex nodematch.Vaccinated absdiff.Age mutual
## Lag 0 1.0000000 1.0000000 1.0000000 1.0000000
## Lag 4096 0.4998500 0.4379654 0.3332490 0.9067160
## Lag 8192 0.3993458 0.3476472 0.2506416 0.8308808
## Lag 12288 0.3575338 0.3064644 0.2116323 0.7646760
## Lag 16384 0.3304047 0.2803422 0.1920482 0.7050629
## Lag 20480 0.3055943 0.2544080 0.1732204 0.6546947
## nodeifactor.Responsibility.TRUE nodeofactor.Responsibility.TRUE
## Lag 0 1.0000000 1.0000000
## Lag 4096 0.4366944 0.4482146
## Lag 8192 0.3279310 0.3403815
## Lag 12288 0.2878303 0.2904896
## Lag 16384 0.2623778 0.2673840
## Lag 20480 0.2441272 0.2473786
## nodeofactor.ProfCat.Auxiliary nodeofactor.ProfCat.Nurse
## Lag 0 1.0000000 1.0000000
## Lag 4096 0.4159969 0.4603978
## Lag 8192 0.3299437 0.3633997
## Lag 12288 0.3005254 0.3239979
## Lag 16384 0.2699148 0.2983247
## Lag 20480 0.2517965 0.2701423
## nodeofactor.ProfCat.Resident Phys.
## Lag 0 1.0000000
## Lag 4096 0.4063175
## Lag 8192 0.3355422
## Lag 12288 0.2977058
## Lag 16384 0.2816249
## Lag 20480 0.2655102
## nodeofactor.ProfCat.Staff Phys.
## Lag 0 1.0000000
## Lag 4096 0.3607954
## Lag 8192 0.2784068
## Lag 12288 0.2473551
## Lag 16384 0.2229325
## Lag 20480 0.1941586
##
## Sample statistics burn-in diagnostic (Geweke):
## Chain 1
##
## Fraction in 1st window = 0.1
## Fraction in 2nd window = 0.5
##
## edges nodeifactor.Vaccinated.TRUE
## 0.49512 0.28636
## nodeofactor.Vaccinated.TRUE nodematch.ProfCat
## -0.09687 0.72709
## nodematch.Unit nodematch.Sex
## 0.60738 0.66915
## nodematch.Vaccinated absdiff.Age
## 0.40455 0.88364
## mutual nodeifactor.Responsibility.TRUE
## 0.38367 1.26964
## nodeofactor.Responsibility.TRUE nodeofactor.ProfCat.Auxiliary
## 1.44799 -0.07918
## nodeofactor.ProfCat.Nurse nodeofactor.ProfCat.Resident Phys.
## 0.47323 -0.34763
## nodeofactor.ProfCat.Staff Phys.
## 1.44970
##
## Individual P-values (lower = worse):
## edges nodeifactor.Vaccinated.TRUE
## 0.6205157 0.7746018
## nodeofactor.Vaccinated.TRUE nodematch.ProfCat
## 0.9228282 0.4671718
## nodematch.Unit nodematch.Sex
## 0.5435996 0.5033996
## nodematch.Vaccinated absdiff.Age
## 0.6858055 0.3768910
## mutual nodeifactor.Responsibility.TRUE
## 0.7012245 0.2042118
## nodeofactor.Responsibility.TRUE nodeofactor.ProfCat.Auxiliary
## 0.1476211 0.9368860
## nodeofactor.ProfCat.Nurse nodeofactor.ProfCat.Resident Phys.
## 0.6360496 0.7281154
## nodeofactor.ProfCat.Staff Phys.
## 0.1471415
## Joint P-value (lower = worse): 0.7824067 .


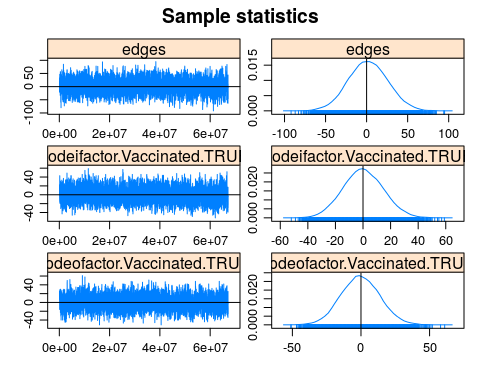

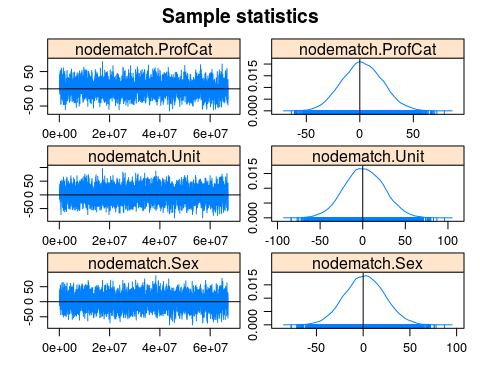

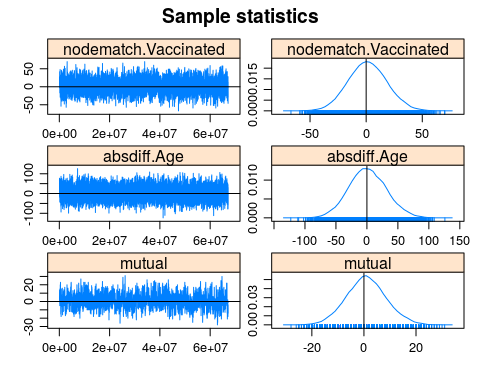

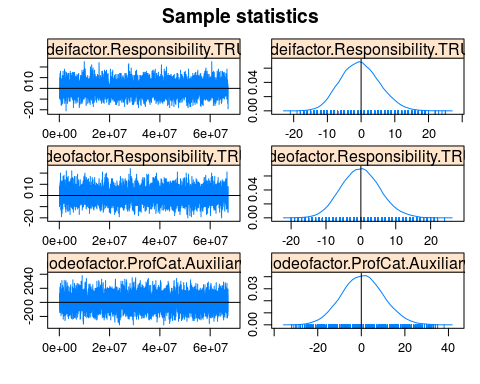

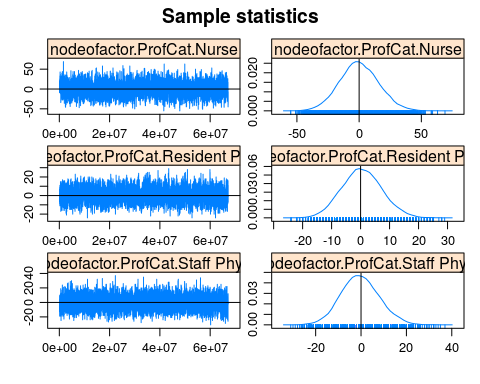


##
## MCMC diagnostics shown here are from the last round of simulation, prior to computation of final parameter estimates. Because the final estimates are refinements of those used for this simulation run, these diagnostics may understate model performance. To directly assess the performance of the final model on in-model statistics, please use the GOF command: gof(ergmFitObject, GOF=~model).

Model 4

## Sample statistics summary:
##
## Iterations = 65536:67170304
## Thinning interval = 4096
## Number of chains = 1
## Sample size per chain = 16384
##
## 1. Empirical mean and standard deviation for each variable,
## plus standard error of the mean:
##
## Mean SD Naive SE
## edges 2.38672 23.639 0.18468
## nodeifactor.Vaccinated.TRUE 1.09094 14.783 0.11549
## nodeofactor.Vaccinated.TRUE 1.04523 14.337 0.11200
## nodematch.ProfCat 1.98065 18.069 0.14116
## nodematch.Unit 2.12622 22.740 0.17766
## nodeifactor.Responsibility.TRUE 0.32727 5.758 0.04499
## nodeofactor.Responsibility.TRUE -0.09619 5.587 0.04365
## nodematch.Sex 1.71350 20.647 0.16130
## nodematch.Vaccinated 2.09113 17.543 0.13705
## absdiff.Age 1.74182 30.044 0.23472
## nodeicov.Age 8.79562 105.069 0.82085
## nodeocov.Age 7.52585 99.687 0.77880
## nodeofactor.ProfCat.Another 0.59546 9.674 0.07558
## nodeofactor.ProfCat.Auxiliary 0.19470 9.558 0.07467
## nodeofactor.ProfCat.Resident Phys. 0.65924 6.791 0.05306
## nodeofactor.ProfCat.Staff Phys. -0.16278 8.455 0.06606
## mutual 0.78558 7.377 0.05763
## Time-series SE
## edges 0.6015
## nodeifactor.Vaccinated.TRUE 0.3937
## nodeofactor.Vaccinated.TRUE 0.3736
## nodematch.ProfCat 0.5772
## nodematch.Unit 0.6017
## nodeifactor.Responsibility.TRUE 0.1473
## nodeofactor.Responsibility.TRUE 0.1527
## nodematch.Sex 0.5944
## nodematch.Vaccinated 0.4476
## absdiff.Age 0.5680
## nodeicov.Age 2.6257
## nodeocov.Age 2.5893
## nodeofactor.ProfCat.Another 0.3029
## nodeofactor.ProfCat.Auxiliary 0.2622
## nodeofactor.ProfCat.Resident Phys. 0.1491
## nodeofactor.ProfCat.Staff Phys. 0.2003
## mutual 0.2918
##
## 2. Quantiles for each variable:
##
## 2.5% 25% 50% 75% 97.5%
## edges -44.0 -14.0 2.0 18.0 49.0
## nodeifactor.Vaccinated.TRUE -27.0 -9.0 1.0 11.0 31.0
## nodeofactor.Vaccinated.TRUE -26.0 -9.0 1.0 11.0 30.0
## nodematch.ProfCat -33.0 -10.0 2.0 14.0 38.0
## nodematch.Unit -42.0 -13.0 2.0 17.0 47.0
## nodeifactor.Responsibility.TRUE -10.0 -4.0 0.0 4.0 12.0
## nodeofactor.Responsibility.TRUE -11.0 -4.0 0.0 4.0 11.0
## nodematch.Sex -38.0 -12.0 1.0 16.0 43.0
## nodematch.Vaccinated -32.0 -10.0 2.0 14.0 37.0
## absdiff.Age -55.9 -18.9 1.4 21.8 61.5
## nodeicov.Age -195.8 -63.1 8.0 79.8 218.8
## nodeocov.Age -187.4 -60.2 6.9 75.4 204.0
## nodeofactor.ProfCat.Another -18.0 -6.0 0.0 7.0 20.0
## nodeofactor.ProfCat.Auxiliary -18.0 -6.0 0.0 7.0 19.0
## nodeofactor.ProfCat.Resident Phys. -12.0 -4.0 0.0 5.0 14.0
## nodeofactor.ProfCat.Staff Phys. -16.0 -6.0 0.0 5.0 17.0
## mutual -13.0 -4.0 1.0 6.0 15.0
##
##
## Sample statistics cross-correlations:
## edges nodeifactor.Vaccinated.TRUE
## edges 1.0000000 0.6907895
## nodeifactor.Vaccinated.TRUE 0.6907895 1.0000000
## nodeofactor.Vaccinated.TRUE 0.6762209 0.6053393
## nodematch.ProfCat 0.7655010 0.5231149
## nodematch.Unit 0.9624150 0.6669555
## nodeifactor.Responsibility.TRUE 0.3026873 0.2430466
## nodeofactor.Responsibility.TRUE 0.2870277 0.1898101
## nodematch.Sex 0.8761275 0.6047665
## nodematch.Vaccinated 0.7290995 0.4113537
## absdiff.Age 0.7609490 0.5164991
## nodeicov.Age 0.9696100 0.6676111
## nodeocov.Age 0.9665768 0.6626829
## nodeofactor.ProfCat.Another 0.4438897 0.3067896
## nodeofactor.ProfCat.Auxiliary 0.4297465 0.3338473
## nodeofactor.ProfCat.Resident Phys. 0.2913329 0.2387126
## nodeofactor.ProfCat.Staff Phys. 0.3793142 0.2650758
## mutual 0.6213129 0.4639254
## nodeofactor.Vaccinated.TRUE
## edges 0.6762209
## nodeifactor.Vaccinated.TRUE 0.6053393
## nodeofactor.Vaccinated.TRUE 1.0000000
## nodematch.ProfCat 0.5198708
## nodematch.Unit 0.6519892
## nodeifactor.Responsibility.TRUE 0.2095717
## nodeofactor.Responsibility.TRUE 0.1970596
## nodematch.Sex 0.5917628
## nodematch.Vaccinated 0.4226321
## absdiff.Age 0.5024770
## nodeicov.Age 0.6513668
## nodeocov.Age 0.6554653
## nodeofactor.ProfCat.Another 0.2930653
## nodeofactor.ProfCat.Auxiliary 0.3824264
## nodeofactor.ProfCat.Resident Phys. 0.2462173
## nodeofactor.ProfCat.Staff Phys. 0.2204512
## mutual 0.4728454
## nodematch.ProfCat nodematch.Unit
## edges 0.7655010 0.9624150
## nodeifactor.Vaccinated.TRUE 0.5231149 0.6669555
## nodeofactor.Vaccinated.TRUE 0.5198708 0.6519892
## nodematch.ProfCat 1.0000000 0.7453266
## nodematch.Unit 0.7453266 1.0000000
## nodeifactor.Responsibility.TRUE 0.2686081 0.2826436
## nodeofactor.Responsibility.TRUE 0.2625558 0.2745512
## nodematch.Sex 0.7012030 0.8435673
## nodematch.Vaccinated 0.5656628 0.7001356
## absdiff.Age 0.5551819 0.7272120
## nodeicov.Age 0.7515876 0.9315539
## nodeocov.Age 0.7589291 0.9312449
## nodeofactor.ProfCat.Another 0.2635774 0.4269934
## nodeofactor.ProfCat.Auxiliary 0.2807386 0.4130743
## nodeofactor.ProfCat.Resident Phys. 0.1473521 0.2717273
## nodeofactor.ProfCat.Staff Phys. 0.2530524 0.3660997
## mutual 0.6013148 0.6379833
## nodeifactor.Responsibility.TRUE
## edges 0.30268735
## nodeifactor.Vaccinated.TRUE 0.24304658
## nodeofactor.Vaccinated.TRUE 0.20957166
## nodematch.ProfCat 0.26860813
## nodematch.Unit 0.28264364
## nodeifactor.Responsibility.TRUE 1.00000000
## nodeofactor.Responsibility.TRUE 0.35137412
## nodematch.Sex 0.23855805
## nodematch.Vaccinated 0.20668674
## absdiff.Age 0.27978938
## nodeicov.Age 0.35617715
## nodeocov.Age 0.32993736
## nodeofactor.ProfCat.Another 0.10285331
## nodeofactor.ProfCat.Auxiliary 0.05809191
## nodeofactor.ProfCat.Resident Phys. 0.05301666
## nodeofactor.ProfCat.Staff Phys. 0.12330738
## mutual 0.22051046
## nodeofactor.Responsibility.TRUE
## edges 0.287027700
## nodeifactor.Vaccinated.TRUE 0.189810132
## nodeofactor.Vaccinated.TRUE 0.197059639
## nodematch.ProfCat 0.262555797
## nodematch.Unit 0.274551163
## nodeifactor.Responsibility.TRUE 0.351374118
## nodeofactor.Responsibility.TRUE 1.000000000
## nodematch.Sex 0.223985591
## nodematch.Vaccinated 0.183489105
## absdiff.Age 0.216824894
## nodeicov.Age 0.313109413
## nodeocov.Age 0.347823439
## nodeofactor.ProfCat.Another 0.133126868
## nodeofactor.ProfCat.Auxiliary -0.006485388
## nodeofactor.ProfCat.Resident Phys. 0.014371423
## nodeofactor.ProfCat.Staff Phys. 0.129740300
## mutual 0.212986794
## nodematch.Sex nodematch.Vaccinated
## edges 0.8761275 0.7290995
## nodeifactor.Vaccinated.TRUE 0.6047665 0.4113537
## nodeofactor.Vaccinated.TRUE 0.5917628 0.4226321
## nodematch.ProfCat 0.7012030 0.5656628
## nodematch.Unit 0.8435673 0.7001356
## nodeifactor.Responsibility.TRUE 0.2385581 0.2066867
## nodeofactor.Responsibility.TRUE 0.2239856 0.1834891
## nodematch.Sex 1.0000000 0.6335080
## nodematch.Vaccinated 0.6335080 1.0000000
## absdiff.Age 0.6630499 0.5616290
## nodeicov.Age 0.8536612 0.7045226
## nodeocov.Age 0.8532830 0.7022895
## nodeofactor.ProfCat.Another 0.3954413 0.3150489
## nodeofactor.ProfCat.Auxiliary 0.4250272 0.3166157
## nodeofactor.ProfCat.Resident Phys. 0.1781879 0.2082334
## nodeofactor.ProfCat.Staff Phys. 0.2700131 0.2768855
## mutual 0.5694604 0.4508658
## absdiff.Age nodeicov.Age nodeocov.Age
## edges 0.7609490 0.9696100 0.9665768
## nodeifactor.Vaccinated.TRUE 0.5164991 0.6676111 0.6626829
## nodeofactor.Vaccinated.TRUE 0.5024770 0.6513668 0.6554653
## nodematch.ProfCat 0.5551819 0.7515876 0.7589291
## nodematch.Unit 0.7272120 0.9315539 0.9312449
## nodeifactor.Responsibility.TRUE 0.2797894 0.3561772 0.3299374
## nodeofactor.Responsibility.TRUE 0.2168249 0.3131094 0.3478234
## nodematch.Sex 0.6630499 0.8536612 0.8532830
## nodematch.Vaccinated 0.5616290 0.7045226 0.7022895
## absdiff.Age 1.0000000 0.7708362 0.7204928
## nodeicov.Age 0.7708362 1.0000000 0.9701837
## nodeocov.Age 0.7204928 0.9701837 1.0000000
## nodeofactor.ProfCat.Another 0.3291309 0.4173902 0.4113855
## nodeofactor.ProfCat.Auxiliary 0.3374938 0.4111241 0.4078118
## nodeofactor.ProfCat.Resident Phys. 0.2033151 0.2313956 0.2026005
## nodeofactor.ProfCat.Staff Phys. 0.2808188 0.3783190 0.3888079
## mutual 0.4037517 0.5924858 0.6194664
## nodeofactor.ProfCat.Another
## edges 0.44388966
## nodeifactor.Vaccinated.TRUE 0.30678959
## nodeofactor.Vaccinated.TRUE 0.29306528
## nodematch.ProfCat 0.26357742
## nodematch.Unit 0.42699341
## nodeifactor.Responsibility.TRUE 0.10285331
## nodeofactor.Responsibility.TRUE 0.13312687
## nodematch.Sex 0.39544133
## nodematch.Vaccinated 0.31504889
## absdiff.Age 0.32913093
## nodeicov.Age 0.41739016
## nodeocov.Age 0.41138548
## nodeofactor.ProfCat.Another 1.00000000
## nodeofactor.ProfCat.Auxiliary 0.01188995
## nodeofactor.ProfCat.Resident Phys. 0.02269620
## nodeofactor.ProfCat.Staff Phys. 0.01770247
## mutual 0.28922938
## nodeofactor.ProfCat.Auxiliary
## edges 0.429746518
## nodeifactor.Vaccinated.TRUE 0.333847327
## nodeofactor.Vaccinated.TRUE 0.382426366
## nodematch.ProfCat 0.280738574
## nodematch.Unit 0.413074291
## nodeifactor.Responsibility.TRUE 0.058091907
## nodeofactor.Responsibility.TRUE -0.006485388
## nodematch.Sex 0.425027221
## nodematch.Vaccinated 0.316615737
## absdiff.Age 0.337493802
## nodeicov.Age 0.411124060
## nodeocov.Age 0.407811769
## nodeofactor.ProfCat.Another 0.011889953
## nodeofactor.ProfCat.Auxiliary 1.000000000
## nodeofactor.ProfCat.Resident Phys. 0.011564753
## nodeofactor.ProfCat.Staff Phys. 0.010414733
## mutual 0.257994214
## nodeofactor.ProfCat.Resident Phys.
## edges 0.291332891
## nodeifactor.Vaccinated.TRUE 0.238712574
## nodeofactor.Vaccinated.TRUE 0.246217300
## nodematch.ProfCat 0.147352106
## nodematch.Unit 0.271727301
## nodeifactor.Responsibility.TRUE 0.053016661
## nodeofactor.Responsibility.TRUE 0.014371423
## nodematch.Sex 0.178187918
## nodematch.Vaccinated 0.208233444
## absdiff.Age 0.203315109
## nodeicov.Age 0.231395603
## nodeocov.Age 0.202600452
## nodeofactor.ProfCat.Another 0.022696204
## nodeofactor.ProfCat.Auxiliary 0.011564753
## nodeofactor.ProfCat.Resident Phys. 1.000000000
## nodeofactor.ProfCat.Staff Phys. -0.004300631
## mutual 0.153513153
## nodeofactor.ProfCat.Staff Phys.
## edges 0.379314161
## nodeifactor.Vaccinated.TRUE 0.265075848
## nodeofactor.Vaccinated.TRUE 0.220451235
## nodematch.ProfCat 0.253052376
## nodematch.Unit 0.366099740
## nodeifactor.Responsibility.TRUE 0.123307384
## nodeofactor.Responsibility.TRUE 0.129740300
## nodematch.Sex 0.270013058
## nodematch.Vaccinated 0.276885466
## absdiff.Age 0.280818771
## nodeicov.Age 0.378319017
## nodeocov.Age 0.388807881
## nodeofactor.ProfCat.Another 0.017702468
## nodeofactor.ProfCat.Auxiliary 0.010414733
## nodeofactor.ProfCat.Resident Phys. -0.004300631
## nodeofactor.ProfCat.Staff Phys. 1.000000000
## mutual 0.211595265
## mutual
## edges 0.6213129
## nodeifactor.Vaccinated.TRUE 0.4639254
## nodeofactor.Vaccinated.TRUE 0.4728454
## nodematch.ProfCat 0.6013148
## nodematch.Unit 0.6379833
## nodeifactor.Responsibility.TRUE 0.2205105
## nodeofactor.Responsibility.TRUE 0.2129868
## nodematch.Sex 0.5694604
## nodematch.Vaccinated 0.4508658
## absdiff.Age 0.4037517
## nodeicov.Age 0.5924858
## nodeocov.Age 0.6194664
## nodeofactor.ProfCat.Another 0.2892294
## nodeofactor.ProfCat.Auxiliary 0.2579942
## nodeofactor.ProfCat.Resident Phys. 0.1535132
## nodeofactor.ProfCat.Staff Phys. 0.2115953
## mutual 1.0000000
##
## Sample statistics auto-correlation:
## Chain 1
## edges nodeifactor.Vaccinated.TRUE
## Lag 0 1.0000000 1.0000000
## Lag 4096 0.4482335 0.4465488
## Lag 8192 0.3613860 0.3400789
## Lag 12288 0.3158380 0.2932842
## Lag 16384 0.2705071 0.2677034
## Lag 20480 0.2515646 0.2453879
## nodeofactor.Vaccinated.TRUE nodematch.ProfCat nodematch.Unit
## Lag 0 1.0000000 1.0000000 1.0000000
## Lag 4096 0.4391082 0.5940613 0.4812622
## Lag 8192 0.3460889 0.4870282 0.3896964
## Lag 12288 0.2990918 0.4339249 0.3405749
## Lag 16384 0.2782597 0.3894207 0.2985553
## Lag 20480 0.2577565 0.3609214 0.2730731
## nodeifactor.Responsibility.TRUE nodeofactor.Responsibility.TRUE
## Lag 0 1.0000000 1.0000000
## Lag 4096 0.4354728 0.4595660
## Lag 8192 0.3281650 0.3449297
## Lag 12288 0.2698668 0.2897484
## Lag 16384 0.2455915 0.2558279
## Lag 20480 0.2266020 0.2460141
## nodematch.Sex nodematch.Vaccinated absdiff.Age nodeicov.Age
## Lag 0 1.0000000 1.0000000 1.0000000 1.0000000
## Lag 4096 0.4763037 0.4539289 0.3300899 0.4430834
## Lag 8192 0.3797823 0.3666494 0.2518881 0.3517594
## Lag 12288 0.3379969 0.3274136 0.1964111 0.3051045
## Lag 16384 0.2914257 0.2824636 0.1651111 0.2629062
## Lag 20480 0.2694014 0.2615502 0.1507219 0.2448237
## nodeocov.Age nodeofactor.ProfCat.Another
## Lag 0 1.0000000 1.0000000
## Lag 4096 0.4621829 0.4569031
## Lag 8192 0.3729962 0.3720243
## Lag 12288 0.3293509 0.3392146
## Lag 16384 0.2849009 0.3017495
## Lag 20480 0.2636770 0.2870393
## nodeofactor.ProfCat.Auxiliary nodeofactor.ProfCat.Resident Phys.
## Lag 0 1.0000000 1.0000000
## Lag 4096 0.4307031 0.3548106
## Lag 8192 0.3376461 0.2682130
## Lag 12288 0.2998473 0.2416001
## Lag 16384 0.2798317 0.2229728
## Lag 20480 0.2550039 0.2113864
## nodeofactor.ProfCat.Staff Phys. mutual
## Lag 0 1.0000000 1.0000000
## Lag 4096 0.3659774 0.9014977
## Lag 8192 0.2852401 0.8204924
## Lag 12288 0.2510162 0.7526868
## Lag 16384 0.2222189 0.6919889
## Lag 20480 0.2132661 0.6396101
##
## Sample statistics burn-in diagnostic (Geweke):
## Chain 1
##
## Fraction in 1st window = 0.1
## Fraction in 2nd window = 0.5
##
## edges nodeifactor.Vaccinated.TRUE
## 1.75170 1.29649
## nodeofactor.Vaccinated.TRUE nodematch.ProfCat
## 1.18627 1.98852
## nodematch.Unit nodeifactor.Responsibility.TRUE
## 1.66767 -0.80643
## nodeofactor.Responsibility.TRUE nodematch.Sex
## -0.78119 1.36723
## nodematch.Vaccinated absdiff.Age
## 1.23866 1.16013
## nodeicov.Age nodeocov.Age
## 1.85698 1.71121
## nodeofactor.ProfCat.Another nodeofactor.ProfCat.Auxiliary
## -0.05655 1.12809
## nodeofactor.ProfCat.Resident Phys. nodeofactor.ProfCat.Staff Phys.
## 2.78132 1.69695
## mutual
## 1.20929
##
## Individual P-values (lower = worse):
## edges nodeifactor.Vaccinated.TRUE
## 0.079826138 0.194806644
## nodeofactor.Vaccinated.TRUE nodematch.ProfCat
## 0.235515949 0.046754625
## nodematch.Unit nodeifactor.Responsibility.TRUE
## 0.095380506 0.419992355
## nodeofactor.Responsibility.TRUE nodematch.Sex
## 0.434690379 0.171553056
## nodematch.Vaccinated absdiff.Age
## 0.215469993 0.245993896
## nodeicov.Age nodeocov.Age
## 0.063313829 0.087043273
## nodeofactor.ProfCat.Another nodeofactor.ProfCat.Auxiliary
## 0.954904445 0.259281584
## nodeofactor.ProfCat.Resident Phys. nodeofactor.ProfCat.Staff Phys.
## 0.005413806 0.089705889
## mutual
## 0.226552253
## Joint P-value (lower = worse): 0.02920402 .


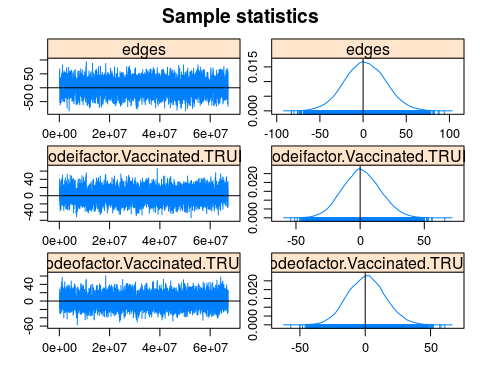

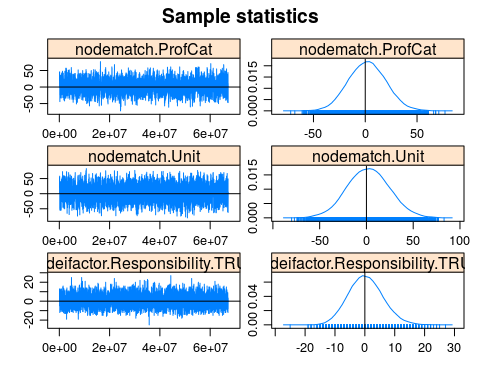

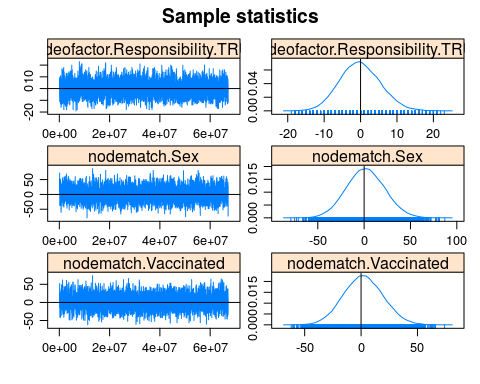

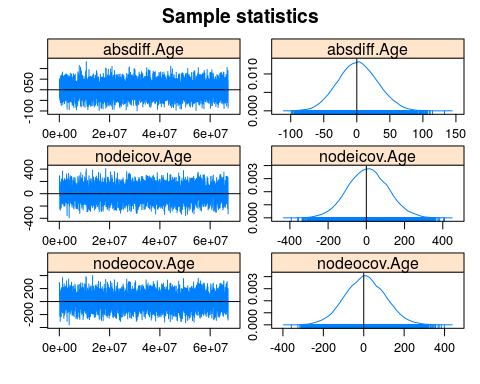

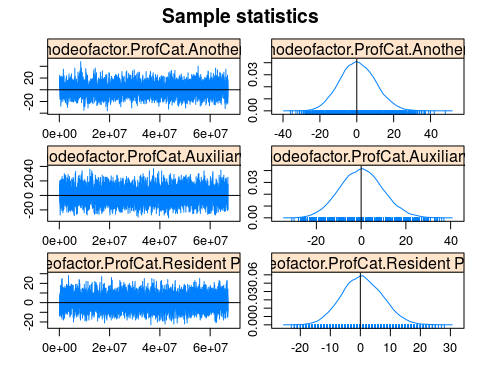

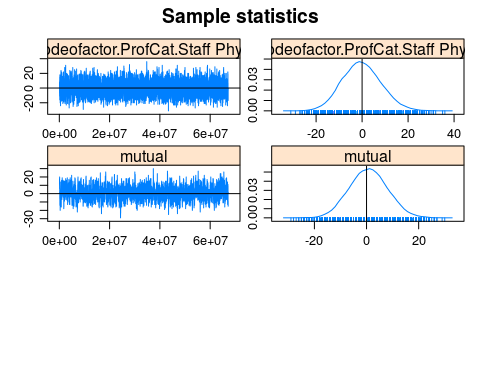


##
## MCMC diagnostics shown here are from the last round of simulation, prior to computation of final parameter estimates. Because the final estimates are refinements of those used for this simulation run, these diagnostics may understate model performance. To directly assess the performance of the final model on in-model statistics, please use the GOF command: gof(ergmFitObject, GOF=~model).
